# Effects of VHA Policy Directive 1163 on Acceptance and Employment Rates for Veterans with Substance Use Disorders Referred to VHA Vocational Rehabilitation

**DOI:** 10.1177/11782218221132397

**Published:** 2022-11-26

**Authors:** Matthew E Sprong, Heaven Hollender, Ashley A Pechek, Kellie Forziat-Pytel, Frank D Buono

**Affiliations:** 1 Department of Veteran Affairs – VA Illiana Health Care System, Danville, IL, USA; 2 VA Edwards Hines Jr. Health Care System, Hines, IL, USA; 3 University of Illinois Springfield, Springfield, IL, USA; 4 Indiana University Purdue University Indianapolis (IUPUI), Indianapolis, IN, USA; 5 Commonwealth University of Pennsylvania (Lock Haven), Lock Haven, PA, USA; 6 Yale School of Medicine, New Haven, CT, USA

**Keywords:** Veterans health, substance-related disorders, rehabilitation, vocational

## Abstract

**Introduction:**

Research has shown that Veterans with Substance/Alcohol Use Disorders (SUDs/AUDs) are at a greater risk for employment-related issues (eg, lower labor force participation rates), and interventions such as Vocational Rehabilitation (VR) have been used as a tool to reduce employment obtainment and maintenance. The purpose of the current study was to evaluate acceptance rates and employment rates at closure for Veterans with SUDs/AUDs prior to the implementation of VHA Policy Directive 1163 (mandated that Veterans are not refused services based on prior or current SUD/AUDs). SUD/AUDs were coded to reflect DSM 5-TR criteria of active use and in-remission.

**Methods:**

Data from a VHA Vocational Rehabilitation program in the Veterans Integrated Service Network 12 network were obtained for the purpose of the current study.

**Results:**

Findings showed that Veterans with AUDs were less likely to be accepted for VR services prior and after implementation of VHA Policy Directive 1163.

**Conclusions:**

When examining active and inactive SUDs/AUDs, findings showed that implementation of VHA Policy Directive 1163 was not effective for Veterans with AUDs. One factor that was not explored but could explain disparities in program acceptance rates is duration of program entry. If a Veteran has a consult placed for VHA Vocational Rehabilitation services, and their program entry date (date accepted) is a significant duration, then perhaps Veterans with active AUDs start drinking again given that they are waiting for vocational assistance. Thus, it would be important to assist Veterans with active AUDs into services in a timely manner (perhaps prior them being discharged from SUD treatment).

## Introduction

Vocational Rehabilitation (VR) is a program that assists people with disabilities in obtaining and maintaining employment that is compatible with their skills, abilities, functional limitations, and interests.^1^ Within the Department of Veteran Affairs Health Care System, the mission of VR is to provide support to Veterans living with mental illness and/or physical impairment and have barriers to securing and maintaining employment.^2^ The Veteran Benefits Administration (VBA) is another service that is separate than the Veteran Health Administration (VHA), in that the focus is on providing Veterans on exploring employment options and addressing the education or training needs (Chapter 31) of Veterans with service-connected disabilities.^3^ There have been many factors identified that impact employment for Veterans, but the focus of the current study is to examine the role that substance use disorders (SUDs) have on VHA VR program acceptance and employment status at closure. Specifically, the current study will include an examination of the implementation of VHA Policy Directive 1163 and its impact for Veterans with SUDs.

### VHA vocational rehabilitation

The VHA VR program was formerly known as Therapeutic and Supported Employment (TSES),^4^ and has also been referred to as Compensated Work Therapy (CWT).^5^ Per Policy Directive 1163, all VHA Medical facilities are required to provide supported-employment (SE) and transitional work (TW), and it is strongly encouraged the community-based employment services (CBES) be offered. Some programs are also encouraged to provide supported education (eg, assisting with the Disability Support Services office), supported self-employment (eg, assisting a Veteran open their own business), and vocational assistance (less intensive program where the Veteran and provider meet on an infrequent basis to assist with minor vocational related issues such as updating a résumé or not requiring intensive job development and placement).

Program eligibility for each program is based on clinical need,^5^ where Veterans with severe mental illness (eg, Bipolar, Active Psychosis) or medical conditions (eg, Traumatic Brain Injury/Spinal Cord Injury) are eligible for the SE program. This programs following the Individual Placement and Support (IPS) evidence-based model, which incorporates 8 CORE functions: (1) open to all who want work, (2) competitive employment emphasis, (3) quick job search, (4) directed job development, (5) the individuals preferences guide decision making, (6) individualized supports that are long-term, (7) treatment integration, and (8) benefits counseling. Specific services may include (1) integration of vocational supports within clinical treatment, (2) assistance with obtaining competitive employment, (3) rapid job search, (4) systematic job development, (5) follow-along supports, (6) focus on Veteran preferences based on their strengths, skills, and interests, and (7) benefits counseling.^6^

Veterans without a severe mental illness or serious medical condition can be deemed eligible for either TW or CBES. TW is a clinical pre-employment VR program in VA medical center and/or business and industry in which Veterans are assigned a real work assignment for a specific and restricted time for the experience.^7^ VHA Directive 1163^5^ specifically references 38 U.S.C. 1718, in that “program participants must not be held or considered employees of the United States for any Purpose. . .and program participants are not subject to criminal background investigations, including fingerprint checks as a condition of acceptance for services” (p.42). Veterans are paid an hourly wage (minimum wage or higher) for the time spent on their assigned worksite. CBES is designed for Veterans with a history of sporadic employment, issues with job retention, or difficulty with job searching.^5^ This program assists Veterans in locating employment and completing the application process, résumé development, practicing for interviews, and other employment-related barriers. The 2019 policy directive mandates that there is a zero-exclusion policy, which indicates that Veterans will receive CBES services regardless of clinical symptoms, legal histories, or other job readiness criteria. However, research has not yet investigated the impact of implementation of Policy Directive 1163 within the Veteran Health Administration (VHA) as it relates to services provided to Veterans with SUDs.

### Substance use, employment, and VHA vocational rehabilitation

An estimated 1.1 million Veterans meet criteria for Substance Use Disorders (SUDs), with prevalence rates between 2016 and 2019 showing alcohol use was 57% to 68% and illicit drugs were 2.7% to 3.5%.^8^ Having an employment emphasis is important when working with Veterans with SUDs since employment status has been shown to be a strong predictor of treatment compliance.^9^ Given that individuals undergoing treatment for alcohol and substance abuse have poor work histories and low employment rates,^10^ the effectiveness that VR can have on a Veteran’s life is well documented.^11-14^ Prior research has shown that employment at closure from VHA Vocational Rehabilitation is impacted by greater treatment intensity (higher weekly mean earnings and longer treatment duration), higher vocational functioning prior to admission (shorter length of time since employed for at least a month), and participation in Transitional Work (TW) within the VA medical facility where they earn tax-free financial incentive to engage in work-therapy.^15^ However, this study did not control for diagnosis (eg, substance use disorders) which is a variable that can impact the findings within this prior study.^8^ Moreover, prior research has shown that military Veterans continue to struggle with addiction even after receiving treatment for substance use disorders, and Veterans that are unemployed/not in the labor force were more likely to relapse than employed Veterans.^16^

Several reasons as to why a Veteran might have difficulty in obtaining employment may include co-occurring substance and psychiatric disorders,^17^ disability,^18^ or limited labor force per residing geographic location. Moreover, Veterans with SUDs may not obtain competitive employment because they endorse high levels of agreement with statements that working (competitive employment) would lead to loss of benefits (eg, supplemental security income, social security disability insurance, or unemployment), and Veterans with SUDs agreed more strongly that they would rather turn down a job offer than lose financial benefits.^19^ Other factors that might impact labor force participation for Veterans with SUDs is because they not being provided services that can help reduce or eliminate employment-related barriers (eg, requesting reasonable accommodations, managing workplace stressors, personal and finance).

VHA Policy Directive 1163^5^ has been updated in August 2019 and has strictly indicated that no discriminatory practices are acceptable for Veterans with substance or alcohol use disorders (AUDs) “regardless of duration of sobriety, routine vocational testing, or required time in a treatment program or clinical services prior to participation in VHA Vocational Rehabilitation” (p. 43). The prior version of the Policy Directive^4^ also stated that “there are no required pathways to participation in any program component such as duration of sobriety, routine vocational testing, or required time in IT prior to placement in another modality” (p. 6). Despite these clear distinctions in the most recent version and prior version of Policy Directive 1163, research has not yet examined the role these policies have in acceptance rates. If disparities do exist for Veterans with SUD/AUD, then further evaluation is needed to determine if it is due to discriminatory practices or other factors where interventions/protocols are needing to be developed or modified.

The current study will focus on evaluating the impact of VHA Policy Directive 1163^5^ on VHA VR program acceptance rates for Veterans with and without SUDs/AUDs. Furthermore,^20^ discussed the benefits of splitting SUD/AUD rather than combing both diagnoses, as findings have shown disparities in employment rates at closure for Veterans with SUD. Furthermore,^20^ found differences in employment status at closure for Veterans with active SUDs compared to Veterans with inactive SUDs (in remission). Although many factors may contribute to these discrepancies, the current study focused on disparities in acceptance rates at program entry. Thus, the purpose of the current study is to (a) determine if barriers related to acceptance into VR services through the Veteran Health Administration are impacted by SUDs/AUDs (both active and inactive), and (b) to study the impact of the implementation of VHA Policy Directive 1163 on acceptance rates, and (c) study the impact of implementation of VHA Policy Directive 1163 on employment rates at closure. The following research questions helped guide the study:

1. How does SUDs/AUDs predict acceptance rates for Veterans referred to a VHA VR program?
2. How does active versus inactive SUDs/AUDs predict acceptance rates for Veterans referred to a VHA VR program?
3. How has the implementation of VHA Policy Directive 1163 predicted acceptance rates for Veterans with SUDs/AUDs?
4. How has the implementation of VHA Policy Directive 1163 predictive acceptance rates for Veterans with active and inactive (in remission) SUDs/AUDs?
5. How has implementation of VHA Policy Directive 1163 predicted employment rates for Veterans with and without SUDs/AUDs, including active and inactive status?

## Methods

### Participants

A total of 2620 Veterans served by a VHA VR program located within the Veterans Integrated Service Network 12 (VISN-12) were obtained for the current study. Only Veterans who were enrolled in the program once during the timeframe of 2016 to 2021 were included in the study due to updated variables/information being included in the intake forms following 2016 (post accreditation obtainment from the Commission on Accreditation of Rehabilitation Facilities (CARF)). However, Veterans on supplemental security income (SSI) or social security disability insurance (SSDI) were also excluded, given that there was a small percentage of program participants that were enrolled in either program, and it would not be appropriate to attempt to control for these variables (SSDI or SSI recipient) within our statistical analysis given the low frequencies.

The final dataset consisted of 923 Veterans with the following SUD/AUD categories. Approximately half the Veterans (n = 503, 54.5%) did not have an AUD or SUD diagnosis, and when examining active versus inactive, descriptive analysis revealed the following frequencies, including: including active SUD (n = 199, 21.6%), inactive SUD (n = 69, 7.5%), active AUD (n = 182, 19.7%), and inactive AUD (n = 103, 11.2%). The average age of the sample was 57.67 (SD = 12.50), and most of the sample were of the male sex (94.4%, n = 873). The majority of the sample had a high school diploma or GED (86.9%, n = 803). Table 1 shows additional demographic information separated by active SUD, inactive SUD, active AUD, inactive AUD, active SUD and AUD, inactive SUD and AUD, and No SUD or AUD.

**Table 1.** Demographic information of study participants.

	Active	Inactive	Active	Inactive	Active	Inactive	No SUD and/or AUD Diagnoses
	SUD		AUD		SUD & AUD		
Gender							
Male	106	28	94	47	72	26	467
Female	2	0	2	0	0	2	36
Race/Ethnicity							
Asian	0	0	1	1	0	0	5
Black (non-Hispanic)	16	6	16	7	8	4	142
Hispanic	2	0	3	0	3	1	19
White (non-Hispanic)	33	7	21	7	9	17	92
Psychiatric disability type							
PTSD	62	3	0	0	0	0	719
Anxiety	0	0	0	0	0	0	640
Depression	57	9	101	37	46	3	670
Adjustment	0	0	0	0	0	0	923
Mood Disorder	0	15	0	1	0	0	907

Frequencies may not equate to 923 participants within the tables above, as participants with 1 active diagnosis (AUD/SUD) may have the other (AUD/SUD) inactive.

### VHA program structure, data source, and procedures

Prior to the completion of any participation within this current study, the primary researcher submitted the proposal to Institutional Review Board (IRB) for the U.S. Department of Veteran Affairs. A letter was provided and indicated that no formal review process was required to use this data because there was no identifying information (also being used for program improvement protocols) and that the researcher was approved for this study. A co-author on the current study received the same response from their University’s IRB, and therefore the data was analyzed to answer the research questions.

VHA Vocational Rehabilitation Services^2^ are offered at every medical center within the 18 Veterans Integrated Services Network (VISN), which is a regional system designed to work together to meet local health care needs.^21^ A program within VISN 12 was utilized in the current study. The program continuously collects information into a data base (Microsoft Access), which includes intake information such as demographic information, treatment team members, educational and employment history, core vocational objectives, family/culture/community background, military service, legal issues, medical and mental health diagnoses and treatment, and employment goals (long-term/short-term). This information is collected via the Computerized Patient Record System (CPRS) and an intake interview (self-report). A veteran may be denied program entry for the following reasons: (1) unable to contact, (2) obtained a job prior to program entry—between consult and initial meeting, (3) pursuing SSI/SSDI, (4) future medical procedure preventing a delay in ability for services, (5) lack of medical clearance from the medical provider, and (6) not interested in obtaining competitive employment—Veteran is referred by their provider but not really interested.

Initially, the data was downloaded into excel, and medical records within CPRS was reviewed to verify information was entered accurately (eg, SUD/AUD status). Active and inactive (in remission) SUD/AUD were reviewed within the problem list and associated medical records to determine if the problem list was accurate (sometimes this information is not updated). For example, recent mental health notes will often times indicate active problems and a biopsychosocial narrative that is not reflected in the problem list. Active and in-active (in remission status) were defined using the DSM 5-TR,^22^ which indicates a person has met criteria for less than 3 months (active usage), and remission status indicates the person who had once met criteria for SUD/AUD has not met criteria for more than 12 months (not counting the presence of cravings). Early remission (3-12 months of not meeting criteria was not categorized within the current study. We verified this information within the medical records, and dichotomized Veterans into several categories, including SUD and AUD, active SUD and inactive SUD, active AUD and inactive AUD. We categorized Veterans into 2 categories as it relates to VHA Policy Directive (pre-implementation, post-implementation). The date of August 19, 2019 was used to split the data, and to avoid crossover effects, we eliminated Veterans from the data analysis inclusion that started prior to the implementation of VHA Policy Directive 1163 but continued to be served after implementation. The program used for the current study incorporated the guidelines from the new Policy Directive on the date of release.

### Data analysis

Prior to analyzing any data [years 2012-2021], the data was limited so that it would reflect Veterans served between 2016 and 2021. The data was coded so that acceptance rates (ie, accepted, not accepted) and employment rates at closure (ie, employed, not employed) were dichotomous. Additionally, the independent variables were dummy coded into a dichotomous variable: active alcohol use disorder (yes/no), inactive alcohol use disorder (yes/no), active substance use disorder (yes/no), inactive substance use disorder (yes/no). Inactive substance/alcohol use disorder are Veterans that have these diagnoses in remission (no symptoms other than cravings for more than 12 months as indicated per the DSM 5 TR). The VHA VR policy directive variable was represented by splitting the data into before and after August 13, 2019, which was the date of official implementation.^5^ Analyses were completed using IBM SPSS Statistics (version 27) software. Descriptive statistics were obtained, and the variables were analyzed within each of the research questions.

For all research questions, binary logistic regressions were performed to evaluate how the outcome variables of acceptance rates and employment rates at closure were impacted by the implementation of VHA Policy Directive 1163. Within SPSS, the variables were inserted as categorical variables within the binary logistic regression analysis feature, and ODD ratios were obtained for significant findings. For the summary of descriptive information contained within the results section prior to the research questions, the equity gap was calculated by taking the difference between each subgroup’s acceptance rate and the overall acceptance rate to show the percentage point gap.^23^

## Results

Descriptive analysis revealed that a total of 363 (39.3%) of Veterans were accepted for VHA VR services between 2016 and 2021. From this total, there were 70 (7.58%) Veterans with active SUDs and 75 (8.13%) with active AUDs that were accepted for VHA VR. Visually, there appears to be an equity gap of 31.72% (SUDs) and 31.17% (AUDs) for acceptance into the program. The descriptive analysis also revealed that approximately 35.18% (SUDs) and 41.21% (AUDs) are accepted for services, when compared to Veterans without these diagnoses. The following research questions provide more specific information as to the significance of substance/AUDs on acceptance into the VHA VR program.

### *Research Question #1*: How does SUDs/AUDs predict acceptance rates for Veterans referred to a VHA Vocational Rehabilitation program?

A binary logistic regression analysis was performed to determine if SUD or AUD significantly predicted program acceptance. Findings showed that SUD (χ^2^[1, N = 923] = 0.557, *P* = .456), AUD (χ^2^[1, N = 923] = 3.430, *P* = .064), and the interaction of SUD/AUD (χ^2^[1, N = 923] = 0.011, *P* = .918) were not significant. When removing the interaction from the logistic regression model, AUD (χ^2^[1, N = 923] = 8.908, *P* = .003, OR = 0.639, and 95% CI = 0.476, 0.857) was significant. See Table 2 for binary logistic regression findings.

**Table 2.** Summary of logistic regression results for acceptance rates (VHA Vocational Rehabilitation Services).

	Estimate	SE	Wald Chi-Square	Sig.	OR	95% Wald Confidence Limits	
Alcohol use disorder (AUD)	0.178	0.239	.557	.456	–	–	–
Substance use disorder (SUD)	−.470	0.254	3.430	.064	–	–	–
AUD × SUD	0.032	0.315	.011	.918	–	–	–

### *Research Question #2*: How does active versus inactive SUDs/AUDs predict acceptance rates for Veterans referred to a VHA Vocational Rehabilitation program?

A binary logistic regression analysis was performed was performed to examine active SUD, inactive SUD, active AUD, inactive AUD on program acceptance rates. Findings showed that the active SUD (χ^2^[1, N = 923] = 2.434, *P* = .119), inactive AUD (χ^2^[1, N = 923] = 0.004, *P* = .949), and inactive AUD (χ^2^[1, N = 923) = 2.256, *P* = .133) were not significant. Veterans with active AUD were less likely to be accepted into the VHA VR program (χ^2^[1, N = 923] = 10.972, *P* < .001, OR = 0.477,and 95% CI = 0.308, 0.739). A separate binary logistic regression was used to measure interactions and simple main effects for the following categories: none, active AUD/SUD (χ^2^[1, N = 636] = 0.004, *P* = .949), inactive AUD/SUD (χ^2^[1, N = 636] = 1.269, *P* = .260), active AUD / inactive SUD (χ^2^[1, N = 636] = 0.825, *P* = .364), and active SUD / inactive AUD (χ^2^[1, N = 636] = 0.125, *P* = .724). See Table 3 for binary logistic regression findings.

**Table 3.** Summary of logistic regression results for program acceptance rates (Research Question 2).

	Estimate	SE	Wald Chi-Square	Sig.	OR	95% Wald Confidence Limits	
Model 1							
active SUD	0.272	0.174	2.434	.119	–	–	–
inactive SUD	−0.017	0.268	0.004	.949	–	–	–
active AUD	−0.740	0.223	10.97	<.001	0.477	0.308	0.739
inactive AUD	−0.266	0.177	2.256	.133	–	–	–
Model 2							
active SUD	0.799	0.659	1.469	.225	–	–	–
inactive SUD	−1.003	0.871	1.327	.249	–	–	–
active AUD	−0.853	0.811	1.108	.293	–	–	–
inactive AUD	−0.933	0.798	1.365	.243	–	–	–
active SUD × active AUD	−0.498	0.577	.747	.388	–	–	–
active SUD × inactive AUD	−0.138	0.391	.125	.724	–	–	–
inactive SUD × active AUD	0.580	0.638	.825	.364	–	–	–
inactive SUD × inactive AUD	0.824	0.731	1.269	.260	–	–	–

### *Research Question #*3: How has the implementation of VHA Policy Directive 1163 predicted acceptance rates for Veterans with SUDs/AUDs?

A binary logistic regression analysis was performed to determine if the implementation of VHA Policy Directive 1163 influenced acceptance rates for Veterans with SUDs, AUDs, and both SUDs/AUDs combined. Findings showed that Veterans with AUDs were less likely to be accepted for program services after implementation of the directive (χ^2^[1, N = 923] = 8.672, *P* = .003, OR = 0.472,and 95% CI = 0.286, 0.778). No other significant findings were shown, including the implementation of policy directive for Veterans with SUDs (χ^2^[1, N = 923] =0.887, *P* = .346) and Veterans diagnoses with both an SUD and AUD (χ^2^[1, N = 636] = 1.878, *P* = .171). A binary logistic regression showed no significant differences in acceptance rates between Veterans with and without a diagnosis of SUD (χ^2^[1, N = 658] = 0.037, *P* = .847), AUD (χ^2^[1, N = 636] = 2.451, *P* = .117), or both an SUD/AUD diagnosis (χ^2^[1, N = 636] = 0.276, *P* = .599) prior to the implementation of VHA Policy Directive 1163. As shown in Table 4, there were no significant findings for acceptance rates after the implementation of VHA Policy Directive 1163 for Veterans with SUDs, AUDs, and both a SUD/AUD diagnoses.

**Table 4.** Summary of logistic regression results for program acceptance rates (Research Question 3).

	Estimate	SE	Wald Chi-Square	Sig.	OR	95% Wald Confidence Limits	
VHA Policy Directive 1163							
SUD	−0.210	0.223	.887	.346	–	–	–
AUD	−0.752	0.255	8.672	.003	0.472	0.286	0.778
SUD × AUD	0.467	0.341	1.878	.171	–	–	–
Prior to VHA Policy Directive 1163							
SUD	−0.257	0.257	.995	0.319	–	–	–
AUD	0.285	0.225	1.599	.206	–	–	–
SUD × AUD	0.202	0.384	.276	.599	–	–	–
Post VHA Policy Directive 1163							
SUD	0.423	0.461	.843	.359	–	–	–
AUD	−0.577	0.456	1.604	.205	–	–	–
SUD × AUD	−0.218	0.578	.143	.706	–	–	–

### *Research Question #*4: How has the implementation of VHA Policy Directive 1163 predicted acceptance rates for Veterans with active and inactive SUDs/AUDs?

A binary logistic regression analysis was performed was performed to examine the impact of implementation of VHA Policy Directive 1163 on program acceptance rates for Veterans with active and inactive SUD/AUD. Findings showed that Veterans with active AUDs were more likely to be accepted prior to implementation (χ^2^[1, N = 923] = 8.529, *P* = .003, OR = 0.496,and 95% CI = 0.310, 0.794). As shown in Table 5, no significant findings were shown for the interaction of policy directive 1163 and active SUD (χ^2^[1, N = 923] = 1.087, *P* = .297), inactive SUD (χ^2^[1, N = 923] = 0.012, *P* = .914), and inactive AUD (χ^2^[1, N = 923] = 0.488, *P* = .485).

**Table 5.** Summary of logistic regression results for program acceptance rates (Research Question 4).

	Estimate	SE	Wald Chi-Square	Sig.	OR	95% Wald Confidence Limits	
VHA Policy Directive 1163							
active SUD	0.272	0.174	2.434	.119	–	–	–
inactive SUD	−0.017	0.268	.004	.949	–	–	–
active AUD	−0.740	0.223	10.97	<.001	.477	0.542	1.084
inactive AUD	−0.266	0.177	2.256	.133	–	–	–
Prior to VHA Policy Directive 1163							
active SUD	0.216	0.215	1.012	.314	–	–	–
inactive SUD	0.047	0.316	.022	.882	–	–	–
active AUD	−0.690	0.260	7.035	.008	0.501	0.301	0.835
inactive AUD	−0.133	0.217	.377	.539	–	–	–
Post VHA Policy Directive 1163							
active SUD	0.443	0.306	2.093	.148	–	–	–
inactive SUD	−0.348	0.555	.392	.531	–	–	–
active AUD	−0.976	0.465	4.406	.036	0.377	0.151	0.937
inactive AUD	−0.573	0.321	3.184	.074	–	–	–

A binary logistic regression showed no significant differences in acceptance rates between Veterans with and without a diagnosis of active SUD (χ^2^[1, N = 618] = 1.012, *P* = .314), inactive SUD (χ^2^[1, N = 618] = 0.022, *P* = .882), and inactive AUD (χ^2^[1, N = 618] = 0.377, *P* = .539). Veterans with active AUDs were less likely to be accepted for services prior to implementation of VHA Policy Directive 1163 (χ^2^[1, N = 618] = 7.035, *P* = .008). As shown in Table 5, there were no significant findings for acceptance rates after the implementation of VHA Policy Directive 1163 for Veterans with active SUD, inactive SUD, active AUD, and inactive AUD.

### *Research Question #5*: How has implementation of VHA Policy Directive 1163 predicted employment rates for Veterans with and without SUDs/AUDs, including active, and inactive status?

A binary logistic regression analysis was performed was performed to examine the impact of implementation of VHA Policy Directive 1163 on employment rates at closure for Veterans with SUDs and/or AUDs. There were no differences in employment rates prior to and after implementation for Veterans with SUDs (χ^2^[1, N = 923] = 3.731, *P* = .053), AUDs (χ^2^[1, N = 923] = 0.262, *P* = .609), or Veterans with both SUD/AUD diagnoses (χ^2^[1, N = 923] = 0.639, *P* = .424). A separate binary logistic regression model was performed with SUD and AUD dichotomized into active and inactive (in-remission). Findings showed that Veterans with active SUD were more likely to exit with employment prior to implementation of VHA Policy Directive 1163 (χ^2^[1, N = 923] = 4.642, *P* = .031, OR = 1.726,and 95% CI = 1.051, 2.836). No significant findings were shown for inactive SUD (χ^2^[1, N = 923] = 0.389, *P* = .533), active AUD (χ^2^[1, N = 923] = 0.142, *P* = .706), or inactive AUD (χ^2^[1, N = 923) = 0.889, *P* = .346). A binary logistic regression showed no significant differences in employment rates at closure prior to the implementation of VHA Policy Directive 1163 for Veterans with SUDs (χ^2^(1, N = 658) = 0.390, *P* = .406), AUDs (χ^2^[1, N = 658] = 0.192, *P* = .541), or Veterans with both SUD/AUD diagnoses (χ^2^[1, N = 658] = 0.374, *P* = .541). As shown in Table 6, there were no significant findings for employment rates at closure before and after the implementation of VHA Policy Directive 1163 for Veterans with SUDs, AUDs, and both a SUD/AUD diagnoses. No significant findings were found for employment rates at closure for Veterans with active SUD, inactive SUD, or inactive AUD (also shown in Table 6). Veterans with active-AUDs were less likely to be accepted for services after implementation of VHA Policy Directive 1163.

**Table 6.** Summary of logistic regression results for employment rates at closure (Research Question 5).

	Estimate	SE	Wald Chi-Square	Sig.	OR	95% Wald Confidence Limits	
VHA Policy Directive 1163							
SUD	−0.307	0.279	1.209	.272	–	–	–
AUD	0.162	0.235	.473	.492	–	–	–
SUD × AUD	−0.353	0.441	.639	.424	–	–	–
Prior to VHA Policy Directive 1163							
SUD	−0.016	0.359	.002	.964	–	–	–
AUD	0.142	0.321	.195	.659	–	–	–
SUD × AUD	−0.345	0.564	.374	.541	–	–	–
Post VHA Policy Directive 1163							
SUD	0.854	0.451	3.509	.061	–	–	–
AUD	0.154	0.366	.177	.674	–	–	–
SUD × AUD	−0.254	0.732	.120	.729	–	–	–
VHA Policy Directive 1163							
active SUD	0.546	0.253	.031	.031	1.726	1.051	2.836
inactive SUD	0.224	0.360	.533	.533	–	–	–
active AUD	−0.267	0.284	.346	.346	–	–	–
inactive AUD	0.093	0.246	.706	.706	–	–	–
Prior to VHA Policy Directive 1163							
active SUD	0.140	0.315	.198	.657	–	–	–
inactive SUD	0.217	0.477	.206	.650	–	–	–
active AUD	−0.324	0.361	.808	.369	–	–	–
inactive AUD	0.180	0.335	.290	.590	–	–	–
Post VHA Policy Directive 1163							
active SUD	1.265	0.437	.379	.004	3.542	1.504	8.339
inactive SUD	0.135	0.580	.054	.816	–	–	–
active AUD	−0.201	0.483	.174	.677	–	–	–
inactive AUD	0.015	0.381	.001	.969	–	–	–

## Discussion

Initial descriptive statistics revealed that Veterans with SUDs and AUDs are less likely to be accepted for VHA VR services compared to people without these diagnoses. This was also evident for Veterans that had active diagnoses (not in remission) for both disorders. Meshberg-Cohen et al^19^ indicated that Veterans with SUDs would turn down a job offer because of fear of losing financial benefits (eg, SSI/SSDI).

### Acceptance rates for veterans referred to a VHA vocational rehabilitation

As evident in research question 1, Veterans with SUD, AUD, and both SUD/AUD diagnoses had no differences acceptance rates for VHA Vocational Rehabilitation services. However, after dichotomizing each of these variables into active and non-active (in-remission), findings in research question 2 showed that Veterans with active AUD were less likely to be accepted into the VHA VR program. Given this significant finding, the implementation of VHA Policy Directive 1163 was examined to determine if differences existed. Findings showed that Veterans with AUD were less likely to be accepted for program services after implementation (research question 3). Again, this variable was examined by dichotomizing the variable into active and in-active (in remission) status, and findings showed acceptance rates were higher prior to the implementation of the policy directive.

The exact reasoning for these findings are unknown, but possible explanations include that (a) perhaps during the consult meeting, these Veterans indicated they were in a position to conduct their own job searches, (b) the length to be placed on a transitional worksite may have influenced relapse potential, and (c) the Veteran re-entered treatment,^16^ or (d) co-occurring psychiatric/medical diagnosis may have influenced additional barriers that prevent acceptance into the program (Veteran was not medically cleared to engage in employment at this time). Other possibilities could be that (a) Veterans thought employment would impact their service-connected disability rating, (b) the COVID-19 pandemic limited employment options and Veterans elected to stay home to prevent contracting the virus, (c) transportation became an issue for attending appointments, or (d) the use of VA Video Connect (VVC) was not the preferred method of care for the Veterans.

### Employment rates for veterans referred to a VHA vocational rehabilitation

Prior research showing the Veterans with SUDs are less likely to be employed^10,17,20^ was consistent with current study findings. The effectiveness that VR can have on a Veteran’s life is well documented,^11-14^ and research is informative in that longer treatment duration, higher vocational functioning prior to admission (shorter period of last employed) and participation in the TW program is beneficial in improving employment outcomes.^15^ However, some explanations to the current study findings may include co-occurring SUD and psychiatric disorders,^17^ disability,^18^ limited labor force per residing geographic location, or Veterans that are unemployed/not in the labor force were more likely to relapse than employed Veterans,^16^ or fear that they would lose financial benefits so Veterans with SUDs will turn down job offers.^19^

The aforementioned findings described are somewhat surprising, given that Veterans with active AUD had disparities in acceptance rates, and results from research question 5 showed disparities in employment rates at closure for Veterans with active SUDs. Further exploration is necessary for both ends of the VHA VR program, as perhaps earlier intervention (VR services when Veterans are enrolled in Mental Health [MH] or SUD counseling) would allow for work-related issues (eg, psychological stress, workplace bullying, issues with reasonable accommodation requests, stigma) to be addressed during treatment. Other suggestions might include having benefit counseling incorporated within early meetings with Veterans to discuss how employment may or may not influence their loss of financial benefits (eg, service-connected disability, SSI/SSDI). It is evident that more research is needed to determine which interventions will have the most impact on decreasing program acceptance and employment status at closure disparities.

### VHA policy directive 1163

Given the several findings from the analyses ran, it appears that the implementation of VHA Policy Directive 1163 [no discriminatory practices are acceptable for Veterans with substance or AUDs “regardless of duration of sobriety, routine vocational testing, or required time in a treatment program or clinical services prior to participation in VHA Vocational Rehabilitation” (p. 43)] may have contributed to the inequities of program acceptance (active AUD) and employment rates at closure active SUD) for Veterans. Since prior research has shown that relapse potential is higher for Veterans with SUDs and are unemployed,^16^ it is necessary for the intervention of VHA VR to be available to all Veterans. Further research should also focus on differences in employment and job retention rates, as the quality of the job may have impact on whether a Veteran retains employment. If the quality of the position obtained is rated differently for Veterans with and without SUDs, this may explain relapse and other issues for Veterans. This will likely result in a policy directive update.

### Limitations

The study is not without limitations. First, this study was used on one VHA VR program due to the difficulty of collecting consistent program evaluation data. Each program collects information pertaining to acceptance dates, program assigned to, closure dates, there are not mandatory policies that dictate what additional variables are collected. For example, some programs may collect data as to whether a Veteran is currently enrolled in substance abuse treatment, whereas other programs may not. Due to the inconsistency in program related data collection processes, it was more appropriate to focus on one program at the current time. Another limitation is data entry errors. Veteran information is entered within the data collection database and verified within the medical management system (CPRS) at the VA, but errors may have still occurred in terms of data entry. Lastly, even though the findings appear promising in terms of the impact of the VHA Policy Directive 1163, other variables may influence the findings, thus impacting the influence of showing causality. In order to demonstrate causality, (1) the cause must come before the effect, (2) the variables must co-vary (be related), and (3) there must not be any confounding variables that could explain the relationship on the dependent variable.^24^ Other variables that might influence acceptance and employment rates at closure from VHA Vocational Rehabilitation may include other factors such as medical disability and co-occurring psychiatric conditions. As such, demonstrating causality was a limitation of the study.

The current study was unable to determine if this was also a factor as to whether Veterans were not accepted into the program (eg, perhaps they disclosed they were not interested in pursuing employment and wanted to be in the TW program that provides tax-free payments). Additionally, the impact of COVID-19 may explain disparities in employment rates at closure during the post-implementation phase of Policy Directive 1163, as a little over 1 year the COVID-19 pandemic changed the landscape of the United States, and Vocational Rehabilitation service delivery models in the public sector^25-30^ and the implementation of telemedicine (VA Video Connect) occurred during the COVID-19 pandemic.^31-33^

## Conclusion and Implications

There were several findings from the current study that showed VHA policy directive 1163 was effective in reducing the acceptance rate inequities for Veterans with and without SUDs/AUDs. This appears promising as VHA Policy Directive 1163^5^ has strictly indicated that no discriminatory practices are acceptable for Veterans with substance or alcohol use disorders (AUDs) “regardless of duration of sobriety, routine vocational testing, or required time in a treatment program or clinical services prior to participation in VHA Vocational Rehabilitation” (p. 43). However, when examining active and inactive SUDs/AUDs, findings showed that implementation of VHA Policy Directive 1163 was not effective for Veterans with AUDs. One factor that was not explored but could explain disparities in program acceptance rates is duration of program entry. If a Veteran has a consult placed for VHA Vocational Rehabilitation services, and their program entry date (date accepted) is a significant duration, then perhaps Veterans with active AUDs start drinking again given that they are waiting for vocational assistance. Thus, it would be important to assist Veterans with active AUDs into services in a timely manner (perhaps prior them being discharged from SUD treatment).

### Future research

There are several directions that future research can focus on for Veterans with SUDs/AUDs receiving VHA VR services. Given these results at the micro-level, conducting a national-level study would add strength and clarity as to whether the policy directive was effective across all VHA VR programs. Other research could include comparing active/inactive (in remission) SUDs/AUDs on closure status (exiting with employment, not exiting with employment) would be important to see the impact of VHA Policy Directive 1163 and COVID-19. Examining co-occurring psychiatric conditions with SUDs/AUDs would determine if additional supports are needed depending on psychiatric groupings (eg, mood, personality). Given that there are 3 primary programs contained within VHA VR (ie, SE, TB, CBES), examining the VHA policy directive and COVID-19 pandemic would be an important component to determine if policy changes are necessary to assist in reducing the inequities (if they exist) within each program for acceptance and closure status.
